# Low pH gel intranasal sprays inactivate influenza viruses in vitro and protect ferrets against influenza infection

**DOI:** 10.1186/1465-9921-8-38

**Published:** 2007-05-17

**Authors:** Paul Rennie, Philip Bowtell, David Hull, Duane Charbonneau, Robert Lambkin-Williams, John Oxford

**Affiliations:** 1 Procter & Gamble Health Sciences Institute, Egham, Surrey, TW20 9NW, UK; 2 Procter & Gamble Health Sciences Institute, Mason, Ohio, USA; 3 Retroscreen Virology Ltd, Centre for Infectious Diseases, Queen Mary School of Medicine and Dentistry, Medical Sciences Building, 327, Mile End Road, London E1 4NS, UK

## Abstract

**Background:**

Developing strategies for controlling the severity of pandemic influenza is a global public health priority. In the event of a pandemic there may be a place for inexpensive, readily available, effective adjunctive therapies to support containment strategies such as prescription antivirals, vaccines, quarantine and restrictions on travel. Inactivation of virus in the intranasal environment is one possible approach. The work described here investigated the sensitivity of influenza viruses to low pH, and the activity of low pH nasal sprays on the course of an influenza infection in the ferret model.

**Methods:**

Inactivation of influenza A and avian reassortment influenza was determined using *in vitro *solutions tests. Low pH nasal sprays were tested using the ferret model with an influenza A Sydney/5/97 challenge. Clinical measures were shed virus, weight loss and body temperature.

**Results:**

The virus inactivation studies showed that influenza viruses are rapidly inactivated by contact with acid buffered solutions at pH 3.5. The titre of influenza A Sydney/5/97 [H3N2] was reduced by at least 3 log cycles with one minute contact with buffers based on simple acid mixtures such as L-pyroglutamic acid, succinic acid, citric acid and ascorbic acid. A pH 3.5 nasal gel composition containing pyroglutamic acid, succinic acid and zinc acetate reduced titres of influenza A Hong Kong/8/68 [H3N2] by 6 log cycles, and avian reassortment influenza A/Washington/897/80 X A Mallard/New York/6750/78 [H3N2] by 5 log cycles, with 1 min contact.

Two ferret challenge studies, with influenza A Sydney/5/97, demonstrated a reduction in the severity of the disease with early application of low pH nasal sprays versus a saline control. In the first study there was decreased weight loss in the treatment groups. In the second study there were reductions in virus shedding and weight loss, most notably when a gelling agent was added to the low pH formulation.

**Conclusion:**

These findings indicate the potential of a low pH nasal spray as an adjunct to current influenza therapies, and warrant further investigation in humans.

## Background

Pandemic influenza, whether from new avian strains or from reassortment within existing strains, is of growing concern [1-3]. If an influenza pandemic of a virulent strain were to emerge, it would rapidly spread around the globe with potential to overwhelm health services. The logistics of mass distribution, coupled with the known limitations of current treatments, mean there is a risk that recommended therapeutic strategies against influenza may leave a significant proportion of the population underprotected [2]. Vaccines are, by definition, one step behind the latest mutation of the influenza virus. Antiviral drugs, such as the neuraminidase inhibitors Oseltamivir and Zanamivir, are effective treatments, provided they can be given to patients early enough [4]. There may be practical limitations to the fast supply of these prescription-only drugs to patients at the optimum disease intervention point during a pandemic. Furthermore, there is the concern over potential for development of viral resistance to these drug interventions. As most patients will deal with influenza at home, a readily available, safe and effective influenza therapy to reduce the severity of the disease, from the early stages of infection, has the potential to be of considerable value in the event of an epidemic or pandemic.

Our studies investigate whether a low pH nasal gel composition could inactivate influenza virus. Some respiratory viruses are known to be sensitive to low pH [5,6]. Rhinoviruses, in particular, are inactivated by acidic conditions, and this is thought to be due to conformational changes in capsid proteins at pH < 6.2, leading to loss of the VP4 subunit [7]. The haemagglutinin structure of influenza virus is known to be pH sensitive and undergoes conformational changes under acidic conditions [8]. The aim of the work reported here was to determine whether a low pH intranasal spray could be effective against influenza virus, initially using *in vitro *solution tests to determine susceptibility of virus to contact with low pH solutions, and then ferret model preclinical studies.

## Methods

### Test formulations

A range of prototype nasal spray formulations was tested (table 1). They were all pH 3.5, buffered, aqueous solutions, based on L-pyroglutamic acid (PCA) with variable secondary acids; ascorbic acid, citric acid, phytic acid and succinic acid. Additionally, some formulations contained zinc acetate dihydrate. Some of the formulations were tested with mucoadhesive gelling agents, Carbopol 980 (Noveon, Cleveland) or hydroxypropylmethyl cellulose (HPMC). Carbopol-containing formulations were not tested *in vitro *due to pipetting difficulties caused by a viscosity increase of the carbomer at the neutralisation stage of the solution tests.

**Table 1:** Composition of formulations tested in solution tests and *in vivo *influenza model

Formulation tested	Code	Solution test	*in vivo *model
PCA/ascorbic acid/phytic acid	PAP	x^a^	
PCA/ascorbic acid/zinc acetate dihydrate	PAZ	x^a^	
PCA/citric acid/phytic acid	PCP	x^a^	x^a^
PCA/ascorbic acid/phytic acid/Carbopol 980	PAPC		x^a^
PCA/ascorbic acid/zinc acetate/Carbopol 980	PAZC		x^a^
PCA/citric acid/phytic acid/Carbopol 980	PCPC		x^a^
PCA/succinic acid/zinc acetate/HPMC	PSZH	x^b^	

^a ^Conducted by Retroscreen Virology, London, UK.

^b ^Conducted by ATS Labs, Eagan, MN, USA

### Virus assay

Influenza A Sydney/5/97 [H3N2] solution tests were conducted by Retroscreen Virology, London, UK. Two hundred microlitres of stock virus at approximately 10^6 ^TCID_50 _in foetal calf serum (FCS) were mixed with 200 μl of test product at 24°C. After 1 minute, the mixture was neutralised by 10-fold dilutions in Minimal Essential Medium (MEM), and assayed for infective virus. The virus was quantified by titration in quadruplicate on Madin Derby Canine Kidney (MDCK) cells in (MEM) with 2.5 ug/ml Tosyl Phenylalanyl Chloromethyl Ketone (TPCK)-treated trypsin, followed by agglutination assay using Turkey Red Blood Cells. Controls without virus were included to test for carry-over cytopathicity of the product into the virus assay. The TCID_50 _was calculated using the Karber equation [9].

Influenza A Hong Kong/8/68 [H3N2] (ATCC VR-544) and avian reassortment influenza solution tests were conducted by ATS Labs, MN, USA. The avian reassortment virus used was A/Washington/897/80 X A Mallard/New York/6750/78 [H3N2] (ATCC VR-2072), prepared originally by Murphy et al (10). Five hundred microlitres of stock virus at approximately 10^5^–10^6 ^TCID_50 _in FCS were mixed with 4.5 ml of test product at 24°C. After 1 minute, the mixture was neutralised by 10-fold dilutions in Minimal Essential Medium (MEM), and assayed for infective virus in quadruplicate, on monolayers of Rhesus Monkey Kidney cells (RMK) with MEM supplemented with 2% heat inactivated fetal bovine serum (FBS). Controls without virus were included to test for carry-over cytopathicity of the product into the virus assay.

### In vivo influenza studies

Female ferrets (either albino or fitch), approximately 6 months old, and body weight 700–800 g, were obtained from Highgate Farm, Market Rasen, UK. Animals were identified by an electronic chip inserted under the skin. They were maintained under controlled diet (Diet F; Special Diet Services, Witham, UK). Prior to the study, blood samples were taken from the animals and a haemagglutinin inhibition assay was performed against Influenza A/Sydney/5/97 to confirm seronegativity to the virus strain. All animal work was conducted in accordance with UK Home Office guidelines. In addition, a thorough review of alternatives was conducted, in line with Procter and Gamble policy of humane treatment and commitment to refinement, reduction and replacement of animal models. In this case there were no viable alternatives nor existing research.

The challenge virus was Influenza A Sydney/5/97 [H3N2], obtained as an allantoic stock from the Retroscreen repository (Retroscreen Virology, London, UK). It was prepared as a 10^3.25 ^TCID_50_/0.1 ml stock in Phosphate buffered saline (PBS). It was administered intranasally to the animals using a pipette. Treatment products were filled into Valois VP7 nasal pump sprays, dosing 100 ml.

### Treatments

The first study was conducted with 24 ferrets, divided into 4 groups of 6 animals (table 2).

**Table 2:** Assignment of animals to treatment and control groups

Ferret assignment	n	Day 0	5 min	Day 1	Day 2	Day 3	Day 4	Day 5	Day 6
Group 1	6	virus challenge	0.1 ml PAPC	0.1 ml PAPC	0.1 ml PAPC	0.1 ml PAPC	0.1 ml PAPC	0.1 ml PAPC	0.1 ml PAPC
Group 2	6	0.1 ml PAPC	Virus challenge						
Group 3	6	virus challenge	0.1 ml PAZC	0.1 ml PAZC	0.1 ml PAZC	0.1 ml PAZC	0.1 ml PAZC	0.1 ml PAZC	0.1 ml PAZC
Group 4 control	6	0.1 ml PBS	Virus challenge						

Group 1 was challenged with 0.1 ml of influenza virus stock per nostril on day 0, and received 0.1 ml of PAPC nasal spray per nostril 5 minutes later. The animals subsequently received a once-daily intranasal dose of test formulation from day 1 to day 6.

Group 2 received a pre-infection application of 0.1 ml per nostril of PAPC nasal spray, followed by virus challenge 5 minutes later.

Group 3 had the same post-infection regime as group 1, with nasal spray PAZC.

Group 4 was a control group. On day 0, they received an intranasal dose of 0.1 ml PBS per nostril, followed by virus challenge 5 minutes later.

A second study was conducted with 18 ferrets, divided into 3 groups of 6 animals (table 3). The purpose was to determine whether addition of a mucoadhesive polymer (Carbopol 980) affected efficacy of the low pH spray.

**Table 3:** Assignment of animals to treatment and control groups

Assignment	n	Day 0	5 min	Day 1	Day 2	Day 3	Day 4	Day 5
Group 1 Control	6	virus challenge	0.1 ml PBS	0.1 ml PBS	0.1 ml PBS	0.1 ml PBS	0.1 ml PBS	0.1 ml PBS
Group 2	6	virus challenge	0.1 ml PCPC	0.1 ml PCPC	0.1 ml PCPC	0.1 ml PCPC	0.1 ml PCPC	0.1 ml PCPC
Group 3	6	virus challenge	0.1 ml PCP	0.1 ml PCP	0.1 ml PCP	0.1 ml PCP	0.1 ml PCP	0.1 ml PCP

Group 1 received 0.1 ml of stock influenza virus per nostril on day 0. Five minutes later, they received 0.1 ml per nostril of Carbopol 980 gel nasal spray PAPC. The animals subsequently received once-daily 0.1 ml intranasal administrations of the test formulations from day 1 to day 5.

Group 2 had the same administration regime as group 2, and received non-mucoadhesive spray PCP.

Group 3 was a control group. They had the same administration regime as the treatment groups, and received 0.1 ml of PBS per nostril. The animals subsequently received once-daily administrations of 0.1 ml PBS from day 1 to day 5.

In both studies, the animals were monitored daily for clinical symptoms; fever (by rectal temperature), weight change, and nasal washes were conducted to estimate virus shedding. The nasal washes were performed under anaesthesia by instillation of 1.0 ml of PBS into each nostril and collection of aspirated fluid. Haemagglutinin assay on MDCK cells was used to determine virus titre in the nasal wash samples.

### Statistical analysis

For the virus data, ANalysis Of VAriance (ANOVA) methods were applied. For temperature and body weight measures, the readings at day 0 were used as a baseline covariate in ANalysis of COVAriance (ANCOVA). Model diagnostics were applied to check the ANOVA and ANCOVA model assumptions. Adjustments for multiple treatment comparisons were made (Sidak) and testing was performed at the 10% significance level.

## Results

**Figure 1:**
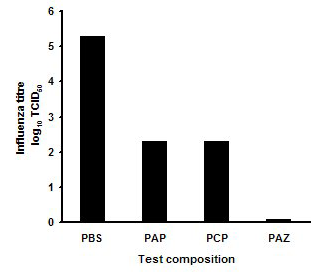
Log reduction in Influenza A titre after 1 min exposure to three pH 3.5 nasal spray compositions in solution test versus a phosphate buffered saline control. PBS: Phosphate buffered saline, pH 7.0. PAP: PCA/ascorbic acid/phytic acid, pH 3.5. PCP: PCA/citric acid/phytic acid, pH 3.5. PAZ: PCA/ascorbic acid/zinc acetate, pH 3.5

All low pH compositions tested rapidly inactivated Human Influenza A. In the first series of experiments (fig 1), the PBS control level of virus was 10^5 ^TCID_50_. Compositions PAP and PCP reduced virus titre by about 3 log cycles with one minute exposure; whereas, with the zinc acetate composition, PAZ, there was no recovered virus, indicating at least 5 log cycles reduction versus control.

**Figure 2:**
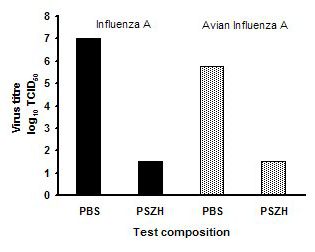
Log reduction in Influenza A and avian Influenza titres after 1 min exposure to a pH 3.5 nasal gel spray composition in solution test versus a phosphate buffered saline control. PBS: Phosphate buffered saline, pH 7.0. PSZH: PCA/succinic acid/zinc acetate/hydroxypropylmethyl cellulose, pH 3.5.

In the second series of experiments with formula PSZH (fig 2), there was no detectable Influenza A or Avian influenza after 1 min exposure, indicating 6 log cycle and 5 log cycle reductions respectively versus controls.

Ferrets infected with influenza develop a self-limited disease with signs similar to those of human influenza [11]. Typically these are; fever, nasal symptoms, general lethargy and decreased rate of weight gain. In an experimental model, the signs usually peak at 48 hours after initial virus challenge. This coincides with an increase in infectious virus shedding and the number of inflammatory cells detected in nasal lavage samples.

**Figure 3:**
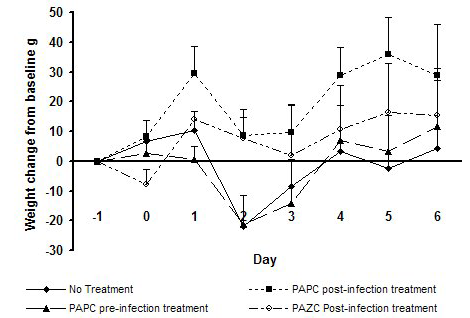
Mean body weight change of ferrets in study 1, challenged with influenza A following pre or post treatment with pH 3.5 gel nasal spray compositions. PAPC: PCA/ascorbic acid/phytic acid/Carbopol 980, pH 3.5. PAZC: PCA/ascorbic acid/zinc acetate/Carbopol 980, pH 3.5. Statistical analysis was performed with ANCOVA, using the day 0 body weight readings as a baseline covariate. Peak day 2 PAPC with post-challenge dosing, difference vs PBS control, p = 0.097. Peak day 2 PAPC with pre-challenge dosing, difference vs PBS control, not statistically significant. Peak day 2 PAZC with post-challenge dosing, difference vs PBS control, p = 0.009

In the first study, virus shedding in the control group peaked at 10^3.1^TCID_50 _at 48 hours. None of the active sprays significantly reduced virus levels. The PAZC spray group showed a 0.6 log TCID_50 _lower virus titre vs control, but this did not reach statistical significance (p = 0.994). There was an increase in mean body temperature of 2°C at 48 hours versus baseline in the PBS spray control group. None of the active sprays significantly reduced febrile response. The PAZC spray group showed a 0.7°C lower mean body temperature, but this did not reach statistical significance (p = 0.467). The mean body weight of the control group dropped by 20 g at 48 hours versus baseline (fig 3). In contrast, animals that were administered with PAZC or PAPC spray once daily after virus challenge showed a significantly reduced weight loss vs control (p = 0.009 and 0.097 respectively). The group with a pre-infection PAPC treatment regime showed a similar weight loss versus control. This group showed a significantly greater weight loss versus PAZC spray group (p = 0.016).

**Figure 4:**
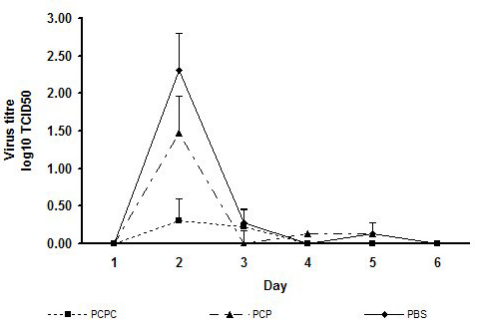
Mean viral titres in ferret nasal washes in study 2, following challenge with Influenza A and treatment with pH 3.5 nasal spray compositions with or without Carbopol 980 gel. PBS: Phosphate buffered saline, pH 7.0. PCPC: Mucoadhesive formula PCA/citric acid/phytic acid/Carbopol 980, pH 3.5. PCP: Non-mucoadhesive formula PCA/citric acid/phytic acid, pH 3.5. Statistical analysis was performed with ANOVA. Peak day 2 PCPC difference vs PBS control, p = 0.026. Peak day 2 PCP difference vs PBS control, not statistically significant.

**Figure 5:**
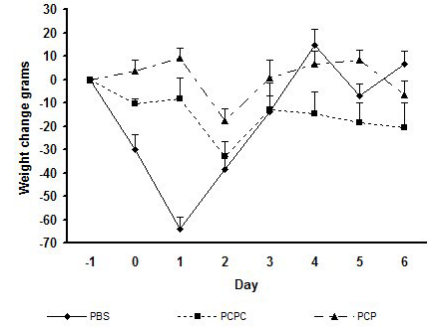
Mean ferret body weight change in study 2, following challenge with Influenza A and treatment with pH 3.5 nasal spray compositions with or without Carbopol 980 gel. PBS: Phosphate buffered saline, pH 7.0. PCPC: Mucoadhesive formula PCA/citric acid/phytic acid/Carbopol 980, pH 3.5. PCP: Non-mucoadhesive formula PCA/citric acid/phytic acid, pH 3.5. Statistical analysis was performed with ANCOVA, using the day 0 body weight readings as a baseline covariate. PCPC difference vs PBS control: Treatment day 1 p = 0.0013, Treatment day 2 p = 0.016. PCP difference vs PBS control, Treatment day 1 p = 0.003, Treatment day 2 p = 0.083.

In the second study, shed virus in nasal lavage samples peaked at 10^2.5^TCID_50 _in the control group at 48 hours (fig 4). In the same group, fever peaked at 0.9°C above baseline at 48 hours after challenge. The control animals lost weight vs baseline (mean -65 g at 24 h and -40 g at 48 hours). The mucoadhesive PCPC spray group showed a mean 2 log TCID_50 _lower virus shedding versus control (p = 0.026). The non-mucoadhesive PCP spray group shed virus titre was not significantly different vs control. The body temperature of the PCPC group at 48 hours was significantly lower versus the PCP group (p = 0.013), but it did not reach statistical significance versus control (p = 0.166). Weight loss was significantly lower in both treatment groups versus control on days 1 and 2. (fig 5). Overall, the non-mucoadhesive spray was less effective than the mucoadhesive spray. It did not reduce fever on peak day 2, nor did it reduce shed virus titre.

## Discussion

Both Influenza A and Avian A were rapidly inactivated by contact with compositions of pH 3.5. Our previous work has shown that pH values close to 3.5 can be achieved in the human nasal cavity, including the nasopharynx region, following administration of a pH 3.5 buffered nasal spray [12]. Infection by enveloped viruses involves fusion of viral and host cell membranes as a prelude to transfer of viral genetic material into the cell [13]. Virus particles are incorporated into endosomes where low pH causes the haemagglutinin to structurally rearrange its shape and activate its fusion potential [8,14]. Haemagglutinin is also responsible for binding influenza viruses to their sialylated cell-surface receptors, so it is conceivable that premature exposure of virus to low pH in the extracellular environment might induce conformational changes to glycoprotein spikes on the virus surface, thereby interfering with binding to the cell. Low pH aggregation of ribonucleocapsids has been reported [15].

The two ferret model studies showed that topical administration of a low pH intranasal spray at the early stage of an influenza infection could reduce the severity of the disease. There was a consistent reduction in weight loss when the spray was administered shortly after virus challenge. It remains to be seen whether the products would be as effective if the spray was administered later in the disease cycle. The observed efficacy is unlikely to be attributable to inactivation of the virus challenge dose before the infection process had begun. Virus was shed throughout the studies by animals in all treatment groups, albeit at lower titres than control groups. This indicates that the initial challenge virus dose was not completely inactivated by the first treatment.

The second study showed that inclusion of a mucoadhesive gel improved the efficacy of the nasal spray. This increased effect may have been due to a coating action on the mucous membranes and an increase in nasal retention. There is a precedent in the field of allergic rhinitis, where application of cellulose powder may reduce hay fever symptoms, presumably by a physical barrier action [16]. A limitation of nasal delivery is the relatively short product retention time in the nose due to mucociliary clearance. The normal residence time of nasally administered solutions in humans is around 12–15 min [17]. The results from the ferret experiments are encouraging since the product could only be applied once a day due to the constraints of anaesthetisation. A higher frequency of dosing might have delivered greater reductions in virus titre.

The efficacy of a low pH topical nasal spray against naturally acquired human influenza remains speculative, especially in light of the paucity of knowledge on the role of nasal infection in the transmission of influenza [18]. Hayden et al [19] showed that intranasal application of a neuramidase inhibitor was effective in a human experimental influenza model. It is unlikely that the low pH action or the antiviral effects of any of the ingredients in the formulations tested in this report would have a systemic action. The formulation is most likely to work topically against extracellular virus in the nasal cavity.

There is evidence that many influenza infections start with cold-like nasal symptoms then spread to the lower airway, whilst others may directly infect the lower airway first [18]. The relative rates of infection by these routes are not known. Hand transmission is believed to play an important role in influenza infection [20], and since the point of entry for the hand route is self-inoculation of the eyes or nose, then a topical nasal spray that delivered an active to the nasopharynx might have a role to play in reducing cross infection. The potential benefits of this approach are likely to be limited to the early stages of an influenza infection, where it could potentially slow the progression of the disease.

The non-specificity of low pH for inactivation of respiratory viruses means that this approach may be less prone to resistance development than current antiviral drugs. The action of the acids is likely to be at multiple points on the virus surface.

## Conclusion

We have demonstrated that low pH nasal sprays can inactivate influenza virus, provided they make contact with the virus. The action is rapid and non specific. Administration of low pH compositions to ferrets has shown that they can influence the course of an experimental influenza infection, with important reductions in severity of the disease. If human influenza benefits were proven, the non-drug nature of the approach means that it might be more readily available to the population at an early stage of infection than current therapies. We conclude that low pH gel nasal sprays are a novel approach to treatment of respiratory virus infections, and that they should be investigated further for the prophylaxis and treatment of early influenza in humans.

## Competing interests

PR, PB and DH are employees of the Procter & Gamble Company which markets respiratory health care products. RL-W and JO are directors of Retroscreen Virology Ltd which provides virus testing services.

## Authors' contributions

PR conceived the study idea and drafted the manuscript

PB conducted the statistical analysis

DH aided the study design and drafting of the manuscript

DC designed the in vitro virology tests

JO and RL-W designed and executed the animal model studies

All authors read and approved the final manuscript.
